# Pulsed Light Synthesis of High Entropy Nanocatalysts with Enhanced Catalytic Activity and Prolonged Stability for Oxygen Evolution Reaction

**DOI:** 10.1002/advs.202300426

**Published:** 2023-04-23

**Authors:** Ali Abdelhafiz, A. N. M. Tanvir, Minxiang Zeng, Baoming Wang, Zhichu Ren, Avetik R. Harutyunyan, Yanliang Zhang, Ju Li

**Affiliations:** ^1^ Department of Nuclear Science and Engineering Massachusetts Institute of Technology Cambridge MA 02139 USA; ^2^ Department of Aerospace and Mechanical Engineering University of Notre Dame Notre Dame IN 46556 USA; ^3^ Department of Materials Science and Engineering Massachusetts Institute of Technology Cambridge MA 02139 USA; ^4^ Honda Research Institute USA, Inc. San Jose CA 95134 USA; ^5^ Department of Chemical Engineering Texas Tech University Lubbock Texas 79409 USA

**Keywords:** green hydrogen production, high‐entropy oxides (HEO), high‐entropy oxyhydroxides (HEOH), high‐throughput synthesis, intense pulse light, noble metal‐free catalysts

## Abstract

The ability to synthesize compositionally complex nanostructures rapidly is a key to high‐throughput functional materials discovery. In addition to being time‐consuming, a majority of conventional materials synthesis processes closely follow thermodynamics equilibria, which limit the discovery of new classes of metastable phases such as high entropy oxides (HEO). Herein, a photonic flash synthesis of HEO nanoparticles at timescales of milliseconds is demonstrated. By leveraging the abrupt heating and cooling cycles induced by a high‐power‐density xenon pulsed light, mixed transition metal salt precursors undergo rapid chemical transformations. Hence, nanoparticles form within milliseconds with a strong affinity to bind to the carbon substrate. Oxygen evolution reaction (OER) activity measurements of the synthesized nanoparticles demonstrate two orders of magnitude prolonged stability at high current densities, without noticeable decay in performance, compared to commercial IrO_2_ catalyst. This superior catalytic activity originates from the synergistic effect of different alloying elements mixed at a high entropic state. It is found that Cr addition influences surface activity the most by promoting higher oxidation states, favoring optimal interaction with OER intermediates. The proposed high‐throughput method opens new pathways toward developing next‐generation functional materials for various electronics, sensing, and environmental applications, in addition to renewable energy conversion.

## Graphical abstract

Diagram showing high‐throughput synthesis of high entropy alloys/oxides (HEOs) nanoparticles, using intense pulsed light. Upon photon absorption by the substrate, temperature rises within a few milliseconds, converting mixed precursors to HEOs nanoparticles. Activity toward oxygen evolution reaction is compared to commercial IrO_2_ catalyst.

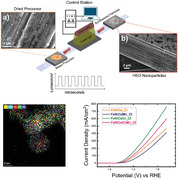

## 1 Introduction

Hydrogen production from water electrolysis is a prominent unit process for net‐zero renewable energy. Electrocatalytic water splitting runs through two half‐cell reactions: hydrogen evolution reaction (HER) and oxygen evolution reaction (OER). For efficient water splitting, one of the current challenges is catalyst design, ensuring low overpotential and long‐term durability.^[^ ^1^, ^2^, ^3^, ^4^ ^]^ In addition, the state‐of‐the‐art catalyst used for OER is noble metal‐based IrO_2_, which adds a burden of high cost toward commercialization. Designing inexpensive, efficient, and durable OER catalysts with a prolonged lifetime is desirable for green hydrogen production.^[^ ^1^, ^3^, ^4^, ^5^, ^6^ ^]^ Transition metals alloying has shown a promising enhancement in water splitting activity in replacing noble metal catalysts.^[^ ^7^, ^8^, ^9^, ^10^, ^11^, ^12^, ^13^ ^]^

High entropy alloy with more than four elements is a unique class of materials with some extraordinary characteristics, e.g., high mechanical strength, tunable magnetic properties, enhanced catalytic activities, etc.^[^ ^14^, ^15^, ^16^, ^17^, ^18^, ^19^, ^20^ ^]^ Exploiting the full potential of HEA materials is suppressed due to limitations of conventional synthesis approaches (e.g., wet chemical or electrochemical syntheses), which tend to be very tedious and complicated.^[^ ^21^ ^]^ Factors such as atomic radii, crystal structure, and valence of the alloying elements affect thermodynamic stability and have to be carefully selected. Otherwise, mixing different elements will result in phase‐segregated compounds instead of achieving a homogeneous mixture of the alloying elements.^[^ ^17^ ^]^ Furthermore, the long processing time (tens of hours to days) are not amenable to high‐throughput accelerated materials discovery. It is thus essential to develop high‐throughput and versatile synthesis approaches that can explore the metastable states of matter.

Recent advances in high‐throughput synthesis, including the carbothermal shock (CTS) approach, have demonstrated the rapid fabrication of high‐entropy alloy (HEA) nanoparticles within seconds,^[^ ^17^, ^22^, ^23^ ^]^ leading to the development of metallic nanoparticles while maintaining precise control over elemental distribution and stoichiometry. CTS operates by rapid Joule heating (up to 1600 °C) and cooling within tens of milliseconds, which empowers kinetics over thermodynamics rules to stabilize metastable phases. Nevertheless, the requirement of an electrically conductive solid substrate is a bottleneck to this process. Herein, we leverage intense pulsed light (IPL) for photon flash synthesis (PFS), which provides a range of fully controlled synthesis parameters (e.g., power density, pulse duration, pulse numbers, and pulse delay) that can dictate the decomposition of metals’ precursors, morphology, and structure of the synthesized high entropy alloy nanoparticles. PFS provides some flexibility in the substrate choices due to the visible photon absorption ability of a diverse range of substrates. This removes the barrier of high electrical conductivity or a solid substrate requirement for the synthesis. PFS is orders of magnitude faster than other techniques utilizing rapid temperature cycling (i.e., repetitive heating and cooling). Moreover, PFS is a noncontact process, making it more industrially scalable, where the fabrication of catalyst electrodes on any light‐absorbing substrates can be done in a single step. While researchers have investigated this rapid fabrication technique for single or binary metal nanoparticle synthesis,^[^ ^24^, ^25^ ^]^ its applicability to produce HEA nanoparticles remains underexplored.

Herein, we report an ultrafast and industrially scalable one‐step synthesis route of non‐platinum‐group‐metal (non‐PGM) high entropy oxides (HEO) nanocatalysts on a carbon fiber paper substrate using PFS. The electrocatalytic activity of synthesized HEO was probed for OER in alkaline aqueous media. Results showed the successful formation of HEO nanoparticles composed of up to five alloying elements, achieving a significantly lower overpotential than IrO_2_, and with prolonged stability at higher current densities (50 and 250 mA cm^−2^) for tens of hours. The nature of the alloying elements (Cr vs Mn) influenced the oxidation state of the active elements (Fe, Ni, and Co) of the synthesized HEO, playing a vital role in optimizing the interaction between reaction intermediates and the catalyst surface. These results indicated that PFS can produce HEO nanoparticles in a high‐throughput manner, compared to tens of hours to days multistep synthesis routes when conventional methods are used (e.g., wet chemical, solid‐state synthesis, chemical vapor deposition). The proposed method can be implemented to fabricate large‐scale electrodes (e.g., 100 cm^2^) of catalyst‐coated gas diffusion electrode (GDE), in a single‐step process, with a production rate of 30 000 electrodes/production line daily. Our approach opens the door for the accelerated discovery of novel functional materials for a wide range of applications, including energy conversion, water treatment, biomedical, and sensing applications.

## 2 Results

### 2.1 Morphology and Structural Analyses

Upon the exposure of the non‐noble metal transition‐metal precursors to IPL, nanoparticles were directly synthesized on carbon fiber paper. The porous carbon paper was selected for 1) strong light absorption ability (low albedo) that promotes the rapid light‐to‐heat conversion; 2) serving as a reducing agent during synthesis. Two sets of samples were fabricated using two different metal precursors salts (i.e., chloride or acetate). Samples are denoted hereon by their elemental composition and based on the salt used (e.g., HEO(FeNiCoMn)_Cl_) for iron‐nickel‐cobalt‐manganese mixture of chloride salts and HEO(FeNiCoCr)_acet_ for iron‐nickel‐cobalt‐chromium mixture of acetate salt). All samples contained exactly the same molarity of total transition‐metal loading, regardless of their composition (i.e., the moles of HEO(FeNiCoMn)_Cl_ = moles of HEO(FeNiCoCr)_acet_), where for each sample all different transition‐metal elements were also equal in molar proportions. **Table** **1** below explains the different annotations used herein to label the samples.

**Table 1:** Samples annotation key

Sample annotation	Made from chloride precursor	Made from acetate precursor
As‐synthesized	HEO(FeNiCoCrMn)_Cl_	HEO(FeNiCoCrMn)_acet_
OER tested	HEOH(FeNiCoCrMn)_Cl_	HEOH(FeNiCoCrMn)_acet_

**Figure 1:**
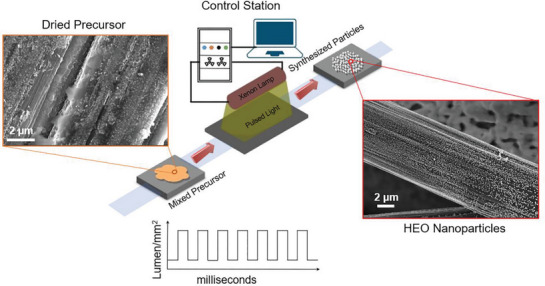
Process diagram for photon‐flash synthesis (PFS) of nanoparticles using an intense pulsed light. Dried salt precursor goes through IPL to synthesize high entropy oxide nanoparticles within milliseconds, as shown by SEM images, left and right images, respectively.

**Figure 2:**
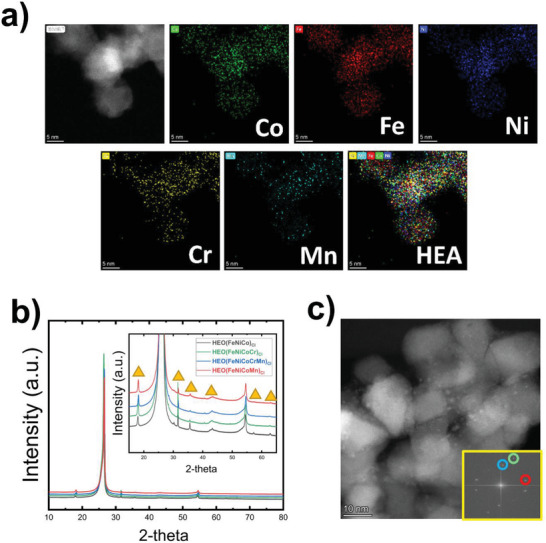
a) Scanning transmission electron microscopy (STEM) imaging of HEO(FeNiCoCrMn)_Cl_ nanoparticles supported on carbon fiber paper (GDE), after decomposition of metal chloride salts mixtures due to exposure to high energy photon pulses. EDX mapping panels show the uniform distribution of elemental composition across the particle. The bottom right panel is an overlay of individual EDX mapping for each element to confirm the successful formation of HEO. b) XRD spectra of ternary, quaternary and quinary HEO NPs showing similar diffraction patterns, suggesting the successful formation of the high entropy phases. Spectra shown in the inset from bottom to top are HEO(FeNiCo)_Cl_, HEO(FeNiCoCr)_Cl_, HEO(FeNiCoCrMn)_Cl_, and HEO(FeNiCoCrMn)_Cl_, respectively. c) STEM image of HEO(FeNiCoCr)_Cl_ HEO cluster of nanoparticles, with an inset showing diffractions at 〈111〉, 〈220〉, and 〈200〉 orientations.

Nanoparticles were synthesized using a computer‐programmed Xenon flash lamp capable of delivering high‐power‐density ultrashort IPL. The schematic in **Figure** **1** illustrates the PFS of high entropy alloy nanoparticles synthesis (the detailed experimental procedure is in the Supporting Information). In a typical process, premixed precursor salts of different metals are first drop‐cast on a carbon fiber paper substrate and dried subsequently. The prepared sample is then irradiated with ultrashort photonic pulses in a flowing N_2_ gas environment. Due to the rapid heating of the substrate caused by the absorption of high‐power density pulsed light, the precursor mixtures were reduced into nanoscale metal particles on the substrate. A uniform conformal coating of the synthesized nanoparticles was achieved throughout the substrate (Figure S1, Supporting Information). The size of the nanoparticles (Feret diameter) varied from tens to hundreds of nanometers (Figure S2, Supporting Information). Scanning electron microscopy (SEM) images showed large nanoparticles of 30–80 nm in diameter. Scanning transmission electron microscopy (STEM) imaging showed that large nanoparticles observed by transmission electron microscopy (TEM) (i.e., secondary particles) are clusters of much smaller primary nanoparticles (5–10 nm in diameter), as shown in **Figure** **2**.

Secondary aggregation of nanoparticles could be a result of the nonuniform thickness of precursor layers deposited by drop casting and the slightly different temperatures across the substrate. The characteristics of the substrate flaws/defects also affect the distribution and morphology of the nanoparticles.^[^ ^17^, ^26^ ^]^ Due to the existence of small wrinkles and larger crater‐like defects on the GDE (Figure S3, Supporting Information), the local temperature of a defect site can be different from the other areas of the substrate. Moreover, larger defects cause the precursor to be accreted irregularly over the substrate, where nanoparticles tend to find the more favorable site of nucleation. Due to mechanical restriction by crater‐like defects, nanoparticles tend to become clustered.^[^ ^26^ ^]^

Atomistic configurations of the synthesized nanoparticles were investigated by STEM, as shown in Figure 2. STEM imaging showed the formation of spherical primary nanoparticles as small as 5–10 nm in diameter, which were observed as a larger secondary particle under SEM. Energy‐dispersive X‐ray (EDX) mapping was performed to investigate the successful incorporation of different alloying elements used in the synthesis protocol and their distribution across the synthesized particles. Figure 2a shows STEM image of HEO(FeNiCoCrMn)_Cl_ sample with a cluster of a few nanoparticles, with a diameter of 5–10 nm each. In addition, Figure 2a shows the results of different EDX elemental mapping, confirming the existence of Fe, Ni, Co, Cr, and Mn elements in the alloyed nanoparticles. All elements were uniformly distributed across the particles, which confirms the successful formation of HEO nanoparticles, which is represented by an overlaid EDX mapping of the different elements at the bottom right panel in Figure 2a. It is worth mentioning that lower intensity and limited distribution were observed for Mn compared to the other elements (i.e., Fe, Ni, Co, and Cr). Mn salt has higher vapor pressure compared to the other elements,^[^ ^27^, ^28^ ^]^ which may render manganese to be less stable than the other elements during the synthesis conditions used herein. Furthermore, X‐ray photoelectron spectroscopy (XPS) analysis showed no signal of Cl, confirming the total decomposition of the metal salts and chlorine atoms leaving as Cl_2_ (gas) during synthesis. Ultrafast heating and cooling cycles enable the successful formation of metastable phases (i.e., HEO) while avoiding phase separation.

X‐ray diffraction (XRD) analysis was performed to investigate the structural features of the as‐synthesized HEO catalysts. Results showed that all the samples maintained spinel oxide crystal structure with dominant peaks shown at 18°, 31°, 35°, 42°, 56°, 62.5° 2‐theta angles. Strong reflections at 27° and 54° correspond to signals from the carbon substrate. The addition of Cr was presumed to cause a shift in the XRD peak due to its larger ionic radius compared to the other elements used. Nevertheless, peak positions for HEO(FeNiCo)_Cl_ and HEO(FeNiCoCr)_Cl_ spectra aligned at the same values. The addition of Cr, as will be shown in later sections, is anticipated to increase the oxidation state of other alloying elements (Fe, Ni and Co), where their ionic radii become smaller. The two competing effects of adding a larger size ion while decreasing the radii of other elements due to oxidation state increase could be canceling each other or generating a minute shift in the peak's positions, which is hard to be observed with XRD resolution. The crystallographic information of the HEO nanoparticles was further investigated using STEM. The crystalline HEO(FeNiCoCr)_Cl_ sample with its fast Fourier transformation is shown in Figure 2c. HEO(FeNiCoCr)_Cl_ samples maintained a spinel oxide crystal structure. Fast Fourier transformation (FFT) image analysis showed the coexistence of multiple crystallographic planes within the HEO nanoparticles (i.e., {111}, {200}, {220}, and {002}), which indicates its polycrystalline nature.

### 2.2 Electrocatalytic Activity toward OER

**Figure 3:**
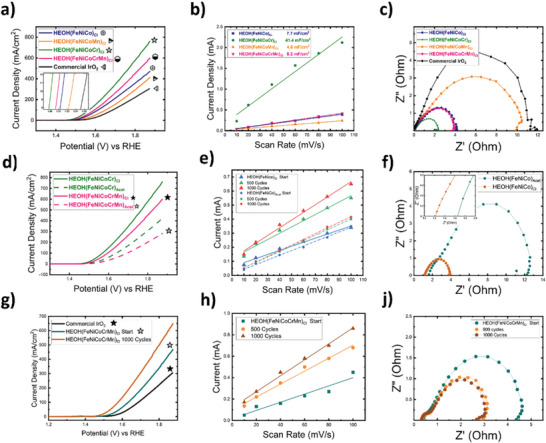
OER performance of different catalysts tested in N_2_ saturated 1 m KOH electrolyte with potential reported versus RHE reference electrode. a) LSV scans for different catalysts synthesized after exposure of metal chloride salts to high‐energy photons compared to commercial IrO_2_. b) ECSA comparison of samples synthesized from chloride salts. c) Nyquist plots showing EIS trends for samples synthesized from chloride salts, with the effect of ohmic resistance deducted to emphasize the effect of different chemical compositions on charge transfer kinetics only. d) LSV comparison of same compositions synthesized using chloride versus acetate salts. e,f) ECSA and EIS data comparing HEOH(FeNiCo)_Cl_ versus HEOH(FeNiCo)_acet_, respectively. g) LSV sweeps of HEOH(FeNiCoCrMn)_Cl_ showing enhancement of activity with CV for 1000 cycles. h,j) showing the effects of CV cycling on ECSA and EIS, respectively, for samples tested in (g). Symbols shown in (a), (d), and (g) are provided to assist with black/white visualization.

Electrocatalytic testing was performed in a three‐electrode setup in a N_2_ saturated 1 m KOH electrolyte. The reference electrode of choice was Hg/HgO with a plastic housing to avoid electrode corrosion during electrochemical testing. **Figure** **3**a shows linear sweep voltammetry (LSV) scans performed at 5 mV s^−1^ scan rate for different chloride‐based salt HEOs. Metal oxide catalysts have been widely reported to attain a hydroxide/oxyhydroxide phase during OER, which has also been reported for similar HEO systems.^[^ ^28^, ^29^, ^30^, ^31^, ^32^ ^]^ Therefore, samples annotation will change hereon to be HEOH for OER‐serviced samples, while HEO is the annotation for as‐synthesized dry samples before electrochemical testing. To evaluate the electrocatalytic performance, two metrics were considered: maximum current density at the end of LSV sweeps, and overpotential at 10 mA cm^−2^. Figure 3a shows superior catalytic activity for all the tested catalysts compared to state‐of‐the‐art commercial IrO_2_ catalyst. The overpotentials calculated at 10 mA cm^−2^ for HEOHs, synthesized from chloride salts, were 250, 268, 279, and 302 mV for HEOH(FeNiCoCr)_Cl_, HEOH(FeNiCoCrMn)_Cl_, HEOH(FeNiCo)_Cl_, and HEOH(FeNiCoMn)_Cl_, respectively. On the other hand, commercial IrO_2_ catalyst showed an overpotential of 317 mV at 10 mA cm^−2^, under the same measurement conditions. To further understand the origin of superior catalytic activity and the effect of chemical composition on activity, electrochemical active surface area (ECSA) is presented in Figure 3b. ECSA was deduced from the electrical double‐layer capacitance (*C*_dl_) derived from cyclic voltammetry (CV) scans performed at different scan rates (i.e., 10, 20, 40, 60, 80, and 100 mV s^−1^). The slope of the linear regression fit represents ECSA for each data set. Results showed that HEOH(FeNiCoCr)_Cl_ catalyst possessed a significantly larger ECSA value, an order of magnitude larger, compared to other HEOH catalysts. HEOH(FeNiCoCr)_Cl_ recorded an ECSA of 41.4 mF cm^−2^. HEOH(FeNiCoCrMn)_Cl_, HEOH(FeNiCo)_Cl_, and HEOH(FeNiCoMn)_Cl_ obtained ECSA values of 8.2, 7.7, and 4.6 mF cm^−2^, respectively. To further understand the performance trends shown by the HEOHs catalysts toward OER, electrochemical impedance measurements (EIS) were performed at 1.6 V versus RHE, as shown in Figure 3c. HEOH catalysts followed the same trend shown in Figure 3a for OER. The smallest EIS was depicted for HEOH(FeNiCoCr)_Cl_ sample, which showed the highest OER activity (i.e., smallest overpotential and maximum current density). Figure 3c shows the Nyquist plot for HEOH catalysts in comparison with commercial IrO_2_ catalyst. HEOH(FeNiCoMn)_Cl_ catalyst showed the largest EIS among the synthesized catalysts, yet, with a smaller value compared to IrO_2_. The fact that all HEOHs catalysts possessed smaller EIS than IrO_2_ highlights faster charge transfer and less‐sluggish reaction kinetics for HEOH samples compared to IrO_2_. In summary, Cr addition making HEOH(FeNiCoCr)_Cl_ showed a superior influence on enhancing OER activity, increasing ECSA by ≈440%, while enhancing the charge transfer kinetics (i.e., smaller EIS) by ≈100%.

The effect of the salt choice (i.e., chloride vs acetate) on OER activity is shown in Figure 3d–f. Figure 3d shows LSV comparison between HEOH(FeNiCoCr)_Cl_ and HEOH(FeNiCoCrMn)_Cl_, and HEOH(FeNiCoCrMn)_acet_ and HEOH(FeNiCoCrMn)_acet,_ where samples synthesized from chloride or acetate salts, respectively. Results showed superior OER activity for HEOHs made from chloride‐based salts samples over their acetate‐based salts counterparts. To further understand the effect of salts’ nature on OER activity, ECSA and EIS measurements were carried out to compare ternary HEOH(FeNiCo)_Cl_ and HEOH(FeNiCo)_acet_, as shown in Figure 3e,f. ECSA data for samples upon testing showed a marginally larger value for HEOH(FeNiCo)_Cl_ compared to HEOH(FeNiCo)_acet_, within a few percent. Nevertheless, HEOH(FeNiCo)_Cl_ and HEOH(FeNiCo)_acet_ samples were tested for 1000 CV cycles (1.3–1.9 V vs RHE) to unravel their performance stability. ECSA trends for both samples showed an increase to larger values, where overpotential after cycling for 1000 cycles became less, as well. HEOH(FeNiCo)_Cl_ showed an increase in ECSA by ≈51 and 88% after 500 and 1000 cycles, respectively. On the other hand, HEOH(FeNiCo)_acet_ showed an increase in ECSA by just 16% after 500 cycles, with a much smaller increase in ECSA after 1000 cycles by an extra 6% (i.e., the total increase in ECSA is 22% after 1000 cycles). This result explains the superior activity for chloride‐based HEOH catalysts over acetate‐based counterparts, where the larger ECSA promotes OER kinetics by providing more active sites to catalyze the reaction per geometric area of the electrode. Nyquist plots in Figure 3f showed a significantly larger EIS value for HEOH(FeNiCo)_acet_ compared to HEOH(FeNiCo)_Cl_, by ≈330%. A larger EIS indicates a more sluggish reaction kinetics for HEOH(FeNiCo)_acet_. In addition, the left intercepts of the semicircle with the *x*‐axis represent the ohmic resistance of the synthesized samples. The inset in Figure 3f shows larger ohmic resistance of HEOH(FeNiCo)_acet_ compared to HEOH(FeNiCo)_Cl_ by 30%. Both samples were tested using the same apparatus and electrical connections, which contribute to the overall ohmic resistance. Thus, the difference in ohmic resistance between HEOH(FeNiCo)_Cl_ and HEOH(FeNiCo)_acet_ can be only attributed to the interfacial interaction between the catalyst nanoparticles and carbon paper support. This indicates that chloride salt favors better dispersion with larger ECSA and more intimate contact with carbon support. These observations shed light on the importance of the precursor salts selection to optimize the catalytic activity.

Cycling stability, where the operating voltage is swept from oxidizing to reducing potentials, is an important metric to assess catalysts’ resilience under reaction conditions. HEOH samples were subjected to CV to investigate their stability for thousands of cycles. As shown earlier in Figure 3e, the ECSA of HEOH(FeNiCo)_Cl_, regardless of the precursor nature (i.e., chloride or acetate), increased with cycling. Figure 3g shows LSV scans of HEOH(FeNiCoCrMn)_Cl_ before and after 1000 cycles, compared to commercial IrO_2_ without cycling. Results showed that HEOH(FeNiCoCrMn)_Cl_ obtained an overpotential of 395 mV compared to 445 mV for IrO_2_ (both before stability cycling) measured at 100 mA cm^−2^. On the other hand, stability cycling enhanced the OER activity of HEOH(FeNiCoCrMn)_Cl_, where overpotential was reduced to 320 mV at 100 mA cm^−2^. In addition, Figure 3h shows that the ECSA of HEOH(FeNiCoCrMn)_Cl_ was monotonically increasing with cycling. ECSA was increased by 59% and 95% after 500 and 1000 cycles. Moreover, the Nyquist plots for HEOH(FeNiCoCrMn)_Cl_ in Figure 3j show a dramatic reduction in EIS after 500 cycles by 44% compared to the EIS value before cycling. Further cycling of HEOH(FeNiCoCrMn)_Cl_ to 1000 cycles, showed an additional, yet slight, reduction in EIS. A similar trend was observed for the ohmic resistance portion of the overall EIS, as shown in Figure S4 (Supporting Information). High entropy samples containing both Cr and Mn (i.e., HEOH(FeNiCoCrMn)_Cl_) showed a much more significant increase in ECSA compared to samples containing either Cr or Mn (i.e., HEOH(FeNiCoCr)_Cl_ or HEOH(FeNiCoMn)_Cl_), after 1000 cycles, as shown in Figures S5–S7 (Supporting Information). This implies that Cr and Mn are much easier to dissolve out of the catalyst compared to Fe, Ni, and Co. Enhancement in OER activity (i.e., reduction in overpotential), and enhancement of the charge transfer kinetics (i.e., decrease of EIS) could be due to surface reconstruction, or leaching of some elements at the utmost catalyst surface layers. Thus, the performance is enhanced due to an enlargement of ECSA or enrichment of the surface chemistry by increasing the population of the active elements (i.e., Fe, Ni, or Co). Similar phenomena have been widely reported in the literature where electrochemical dealloying is a favorable method to boost the activity of electrocatalysts.^[^ ^33^, ^34^, ^35^, ^36^ ^]^ Mn and Cr could be much less stable, where they are easier to evolve to the surface compared to other elements (Fe, Ni, or Co). Hence, more porosity will open up (i.e., higher surface area) or more enhancement of the catalytically active sites of Ni.^[^ ^29^, ^30^, ^31^, ^32^ ^]^ Zhao et al. reported an enhancement of OER activity when Mn content is enriched within the vicinity of Ni, where OH^−^ deprotonation is enhanced.^[^ ^28^, ^37^, ^38^ ^]^

### 2.3 Accelerated Durability Testing (ADT)

**Figure 4:**
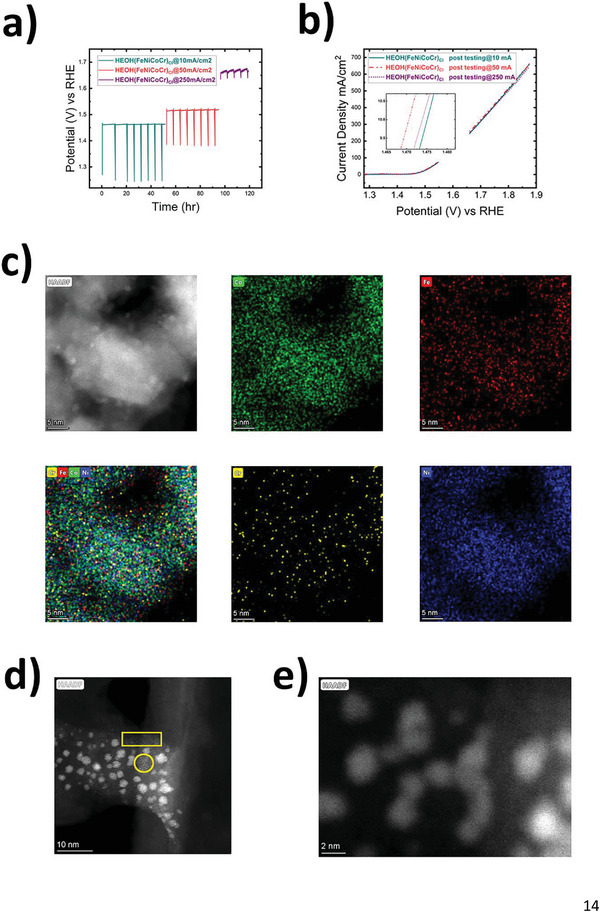
a) Consecutive chronopotentiometry ADT of HEOH(FeNiCoCr)_Cl_ at 10, 50, and 250 mA cm^−2^ for 50, 50, and 20 h, respectively, shown in green, red and purple, from left to right. b) LSV scans after each chronopotentiometry interval, with an inset showing LSV at 10 mA cm^−2^. c–e) TEM images of HEOH(FeNiCoCr)_Cl_. c) TEM/EDX elemental mapping after ADT. d) TEM image showing single atomically dispersed catalyst after ADT, highlighted with yellow circle and rectangle. e) TEM image showing a majority of particles with 2 nm in diameter.

Electrocatalytic activity is very important, as poor catalyst durability has stopped the translation of many laboratory‐scale successes to real applications (e.g., AEMEC). Catalyst durability has to meet extended operation for 100s of hours without noticeable decay in performance. Transition‐metal HEOs synthesized by PFS, with no incorporation of any noble metals (e.g., Pt or Ir) showed excellent activity for OER. Synthesized HEOs are very promising due to their inexpensive elements and the high‐throughput nature of the proposed synthesis protocol, where 1000s of electrodes for MEA can be produced at the same time, compared to conventional methods (e.g., wet chemical synthesis + catalyst coating on GDE) which can produce much fewer. ADT was performed to evaluate the stability of our synthesized HEO catalysts supported on carbon paper. Samples were subjected to the constant current mode (i.e., chronopotentiometry [CP]) while observing the changes in potential, where any positive increase in potential represents a decay in OER performance. **Figure** **4**a shows CP series of tests performed on HEOH(FeNiCoCr)_Cl_ catalyst. ADT protocol was deployed by performing CP at 5‐ or 10‐h intervals, followed by OCV for 30 min and LSV to record the OER performance after each CP step. OCV insertion in the testing protocol was mainly to help relieve the catalyst from accumulated gas bubbles on the surface, which causes a decay in the electrocatalytic activity by blocking the accessibility of electrolyte/catalyst/reactants interface.^[^ ^39^ ^]^ As shown in Figure 4a, samples were tested by a series of successive CP. Initially, samples were tested at CP of 10 mA cm^−2^ for 50 h total (CP^10^), followed by CP at 50 mA cm^−2^ for an additional 50 h (CP^50^), and finally CP at 250 mA cm^−2^ for 20 h (CP^250^). Potential changes at each of the CP steps are shown in Figures S8–S10 (Supporting Information), where the rate of potential change was 0.04, 0.08, and 0.32 mV h^−1^ after CP^10^, CP^50^, and CP^250^, respectively. Figure 4b shows LSV scans taken after the completion of CP^10^, CP^50^, and CP^250^ intervals. The overpotential measured at 10 mA cm^−2^ after CP^10^ for 50 h was 245 mV. After completion of CP^50^ at the 100‐h mark, the overpotential decreased to 240.5 mV. This shows that our HEOH(FeNiCoCr)_Cl_ catalysts’ activity was further enhanced after catalyst stability testing, instead of degrading. Finally, after the completion of CP^250^, the overpotential slightly increased to 244 mV, still better than the starting overpotential before testing or after CP^10^ (i.e., 250 mV). HEOH(FeNiCoCr)_Cl_ catalyst demonstrated tremendous stability after higher current densities before observing any losses in OER performance. It is worth mentioning that the synthesized HEOs presented herein possess two orders of magnitude longer stability compared to state‐of‐the‐art commercial IrO_2_, which did not survive a few hours of testing at 10 mA cm^−2^ where a potential jump was observed, which is an indication of complete IrO_2_ deactivation, as shown in Figure S11 (Supporting Information) and in agreement with other literature studies showing similar very limited stability for IrO_2_.^[^ ^6^, ^7^ ^]^

HEOH(FeNiCoCr)_Cl_ catalyst after ADT testing was characterized by TEM to reveal the chemical and morphological changes, as shown in Figure 4c. EDX mapping showed a significant loss in the signal of Cr, which suggests that Cr was leached away during the electrochemical testing. In addition, Fe signal slightly decreased, but remained evenly distributed across the particle. Both signals from Co and Ni remained intensified. XPS scan for Cr showed no signal at all, which suggests Cr loss from the vicinity of the surface, where weak Cr signal depicted in STEM/EDX was mostly from within the bulk. Further details about the origin of activity enhancement in correlation to the chemistry of HEOs are presented next. Figure 4d,e shows TEM images of HEOH(FeNiCoCr)_Cl_ after ADT. Several spots showed the existence of single‐atom catalysts (highlighted with the yellow circle and rectangle in Figure 4d). Single atoms evolution is a result of bubbles and oxygen leaving the primary particles under OER conditions, which has been widely observed in literature for other perovskites and high entropy oxides systems.^[^ ^28^, ^30^, ^31^ ^]^ Moreover, after ADT the particle size becomes clearly evident to be within 2 nm or smaller. The aforementioned observations of morphological changes, with a tendency to enlarge ECSA, give further explanation of activity enhancement with OER operation. In addition, XRD analysis for samples after ADT, showed similar diffraction spectra to those before ADT (Figure S12, Supporting Information), which infers no significant change in crystal structure took place.

### 2.4 Discussion: Effect of HEO Composition on OER Catalytic Activity

Non‐noble metals showed interesting activity toward catalyzing OER in an alkaline medium. Transition metals’ (e.g., Fe, Ni, Co) catalytic activity is a function of the interaction between the metal surface and reaction intermediates. Metal‐oxygen covalency can be a descriptor to explain the superior activity of transition metals from the poor ones, while assessing the degree of overlap between *d*‐band orbitals of the transition metal and *2p* orbital of oxygen of the reaction intermediates. Increasing the overlap between both orbitals indicates a stronger interaction between oxygen reaction intermediates and transition metal catalyst surface. In this context, it was observed that increasing the oxidation state of the transition‐metal catalyst, leading to less filled *3d* orbital, increases its electronegativity. Hence, the *d*‐band center shifts downwards, leading to more resonance between *3d* orbitals of the transition metal (e.g., Fe, Ni, Co) and *2p* orbitals of the reaction intermediates. As a consequence, OER activity is getting enhanced due to stronger metal–oxygen hybridization. Reported in literature for Li‐Co oxides that when Li was dealloyed, the oxidation state of Co increases, where the activity toward OER increased due to more metal–oxygen orbital overlapping. In addition, Zhu et al. observed a few‐fold enhancement in OER activity for Fe‐containing oxide alloys, through modulating the oxidation state of Fe species, where an increase in Fe oxidation state dramatically enhanced OER activity. Furthermore, increasing the oxidation state of Co tends to strengthen the interaction between Co and oxygen reaction intermediates by enabling the creation of —OOH intermediates at the catalyst surface. Similar observations have been widely shown in literature for perovskites and spinel oxides alloys with multiple metal centers (e.g., Ni, Fe, and Co).^[^ ^7^, ^28^, ^37^, ^38^, ^40^, ^41^ ^]^

**Figure 5:**
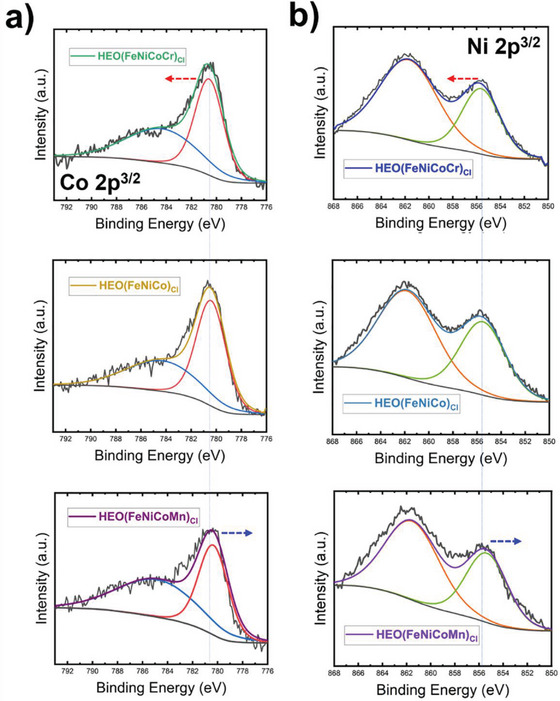
a,b) XPS spectra of Co 2p^3/2^ and Ni 2p^3/2^ photoemissions, respectively, for HEO(FeNiCoCr)_Cl_, HEO(FeNiCo)_Cl_, and HEO(FeNiCoMn)_Cl_, showing peaks compared to HEO(FeNiCo)_Cl_ peak centered by vertical blue line for each case. Shifts to higher and lower BE of HEO(FeNiCoCr)_Cl_ and HEO(FeNiCoMn)_Cl_, indicated by red and blue arrows, respectively.

To reveal the origin of OER catalytic activity enhancement, XPS was used to collect photoemissions from different elements of HEOHs samples. **Figure** **5**a shows Co 2p^3/2^ photoemissions for HEOH(FeNiCoCr)_Cl_, HEOH(FeNiCo)_Cl_, and HEOH(FeNiCoMn)_Cl_ from top to bottom, respectively. HEOH(FeNiCo)_Cl_ at the middle panel can be taken as a reference to unravel the effect of the different alloying elements (i.e., Cr or Mn) on the electronic structure of the active elements (e.g., Co or Ni), in correlation to their corresponding OER activity trend observed earlier (as shown in Figure 3). Co 2p^3/2^ photoemission peaks’ center position is an indication of the change in the oxidation state of Co. Addition of Mn to form HEOH(FeNiCoMn)_Cl_ showed a negative shift of Co 2p^3/2^ to lower binding energies, compared to Co 2p^3/2^ of HEOH(FeNiCo)_Cl_ sample. Co 2p^3/2^ of HEOH(FeNiCoMn)_Cl_ centered at 780.3 eV compared to 780.6 eV for HEOH(FeNiCo)_Cl_ sample. This observation indicates that the addition of Mn tends to decrease the oxidation state of Co, which is expected to show lower activity compared to HEOH(FeNiCo)_Cl_, similar to the trend observed in Figure 3a. This further agrees with the more sluggish kinetics deduced from the larger EIS semicircle as shown in Figure 3c. On the other hand, the addition of Cr induced a shift to higher binding energies of Co 2p^3/2^ photoemissions to center at 780.8 eV. This indicates a significant increase in the oxidation state of Co compared in HEOH(FeNiCoCr)_Cl_ compared to HEOH(FeNiCo)_Cl_ and HEOH(FeNiCoMn)_Cl_ counterparts, which aligns with the superior OER activity trend reported for HEOH(FeNiCoCr)_Cl_ as shown in Figure 3a, with faster electron transfer kinetics as shown with the smallest EIS value in Figure 3c.

Photoemissions of Ni 2p^3/2^ for the different sample sets are shown in Figure 5b. Ni 2p^3/2^ for HEOH(FeNiCo)_Cl_ sample is centered at 855.6 eV. The addition of Mn, forming HEOH(FeNiCoMn)_Cl_, resulted in a shift of Ni 2p^3/2^ to lower binding energies (855.3 eV). This indicates a reduction in the oxidation state of Ni, similar to the observed reduction in the Co oxidation state for the same sample. On the other hand, the addition of Cr to form HEOH(FeNiCoCr)_Cl_ quaternary HEO, showed an opposite trend where photoemissions of Ni 2p^3/2^ shifted to higher binding energies (885.6 eV). Alloying Cr with HEOH(FeNiCo)_Cl_ shows a positive effect on the oxidation state of Ni, which aligns well with the superior electrochemical performance shown earlier. The observations of oxidation state shift and its corresponding effect on OER activity suggest that the nature of the alloying element (Cr or Mn) has more influence in dictating the catalytic activity than increasing the configurational entropy (i.e., increasing the alloying order from ternary HEOH(FeNiCo)_Cl_ to quinary HEOH(FeNiCoCrMn)_Cl_).

**Figure 6:**
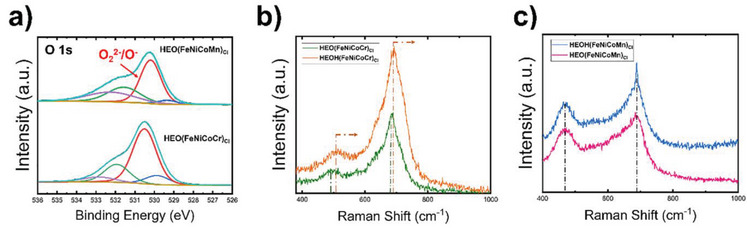
a) XPS of oxygen O 1s spectra deconvoluted into four peaks lattice oxygen of the metal oxide, highly oxygenated species O_2_^2−^/O^−^, surface adsorbed O_2_/OH^−^, and H_2_O adsorbed on the catalyst surface, which are shown in blue, red, green, and magenta, respectively. b,c) Raman analysis for the as‐synthesized catalyst [top spectra of (b) and (c)] and after OCP [bottom spectra of (b) and (c)]. b) Raman spectra for HEO(FeNiCoCr)_Cl_ shown in green, where peaks shift to higher Raman shifts after electrochemical treatment in 1 m KOH (i.e., HEOH(FeNiCoCr)_Cl_), shown in orange. c) Raman spectra of HEO(FeNiCoMn)_Cl_ and HEOH(FeNiCoMn)_Cl_ are shown in magenta and blue, respectively, where no noticeable peak shift was observed after electrochemical treatment.

**Figure** **6**a shows XPS spectra of O 1s photoemissions. O 1s peak can be deconvoluted into four peaks: lattice oxygen of the native HEA metal oxide, highly oxygenated species O_2_^2−^/O^−^, surface adsorbed O_2_/OH^−^, and H_2_O adsorbed on the catalyst surface, which are shown in blue, red, green, and magenta, respectively.^[^ ^42^ ^]^ HEOH(FeNiCoCr)_Cl_ showed enhancement in the peak area of the surface‐adsorbed highly oxygenated species (O_2_^2−^/O^−^) compared to HEOH(FeNiCoMn)_Cl_, which has been ascribed to motivate OER activity at elevated pH values.^[^ ^7^, ^32^, ^42^ ^]^ In addition, the peak assigned to surface adsorbed O_2_/OH^−^ of HEOH(FeNiCoCr)_Cl_ has a larger area compared to HEOH(FeNiCoMn)_Cl_ counterpart, which indicates more accessibility of reaction intermediates to the catalyst for samples containing Cr versus Mn. This further aligns with the ECSA trend observed *C*_DL_ measurements, as shown in Figure 3b. Figure 6b shows Raman spectra of the as‐synthesized HEO(FeNiCoCr)_Cl_ and HEOH(FeNiCoCr)_Cl_ right after exposure to 1 m KOH within a three‐electrode setup for 5 min at the open‐circuit voltage (OCV). The lifetime of hydroxide or oxyhydroxide phases formed during OCV is 1 h. Therefore, Raman analysis on samples exposed to 1 m KOH was conducted in less than 60 min, to trace the samples as close as possible to the nature possessed within the reaction medium. Figure 6c shows Raman spectra of the as‐synthesized HEO(FeNiCoMn)_Cl_ and HEOH(FeNiCoMn)_Cl_, respectively. HEOH(FeNiCoMn)_Cl_ samples has a peak located at ≈480 cm^−1^, and the same peak is observed at a higher wavenumber for HEOH(FeNiCoCr)_Cl_ (≈495 cm^−1^). Moreover, HEOH(FeNiCoCr)_Cl_ samples showed an interesting phenomenon where peaks located at ≈495 and 680 cm^−1^ both shifted to higher Raman shifts after OCV, as shown in Figure 6b. On the other hand, HEOH(FeNiCoMn)_Cl_ samples showed no noticeable peak shift after OCV. Raman shifts to lower wavenumbers have been observed for materials induced with tensile strain. On the contrary, materials subjected to compressive strain have their atomic bond‐bond distance to be shorter. Hence, the Raman response of a compressed material will be observed by a shift in the peak position to higher wavenumbers.^[^ ^43^ ^]^ Strain engineering is a key approach in boosting the electrocatalytic activity for a wide range of reactions, where compressive strain induced on the metal surface due to defects, epitaxial growth or alloying can boost the electrocatalytic activity and stability significantly.^[^ ^35^, ^36^ ^]^ Cr has a larger ionic radius compared to Mn. Evolution of Cr toward the surface under OER condition, before dissolving completely into the electrolyte,^[^ ^28^ ^]^ could induce compressive strain where Raman oscillations shift to higher wavenumbers, and an increase in the catalytic activity of metal adatoms (i.e., Fe, Ni, and Co).^[^ ^35^, ^36^ ^]^

## 3 Conclusion

A rapid processing technique to produce high‐entropy oxide and oxyhydroxide nanoparticles is established, showing the particle morphology, dispersion, and enhanced catalytic performance for electrocatalytic oxygen evolution reaction. Synthesized transition‐metal HEOHs showed improved catalytic activity and stability compared to state‐of‐the‐art IrO_2_ catalysts for catalyzing OER reactions. The incorporation of Cr as an alloying element enhanced the activity of HEOH catalyst, compared to Mn, due to the promotion of higher oxidation state of active species (Ni and Co) and maximizing the adsorption of highly oxygenated species at the catalyst surfaces. Electrochemical cycling showed enhancement in OER activity, supposedly due to leaching out of less stable elements (i.e., Mn and Cr). OER activity enhancement with cycling is owed to the enlargement of ECSA and enhancement of charge transfer kinetics (smaller impedance). HEOH catalysts showed two orders of magnitude prolonged stability compared to commercial IrO_2_, without noticeable decay in OER performance. The IPL technique demonstrated the successful high‐throughput synthesis of various HEOH nanoparticles and provided a prime example of developing compositionally complex nanostructures within a time scale of milliseconds, which opens the door toward accelerated materials discovery for a variety of applications from energy conversion to sensing and water treatment.

## Conflict of Interest

The authors declare no conflict of interest.

## Supporting information

Supporting Information

## Data Availability

The data that support the findings of this study are available from the corresponding author upon reasonable request.
