# Supplementary material for: Pulsed Light Synthesis of High Entropy Nanocatalysts with Enhanced Catalytic Activity and Prolonged Stability for Oxygen Evolution Reaction
Source: Adv Sci (Weinh). 2023 Apr 23;10(18):2300426. doi: 10.1002/advs.202300426 (PMC10288253; doi:10.1002/advs.202300426)
Supplement: Supplementary file 1 — Supporting Information [file ADVS-10-2300426-s001.pdf]

Supplementary Information

Pulsed Light Synthesis of High Entropy Nano-catalysts with Enhanced Catalytic Activity and Prolonged Stability for Oxygen Evolution Reaction

Ali Abdelhafiz[#,1], A. N. M. Tanvir[#,2], Minxiang Zeng[2], Baoming Wang[3], Zhichu Ren[3], Avetik R. Harutyunyan[1,4], Yanliang Zhang*[,2], Ju Li*[,1,3]

[1] Department of Nuclear Science and Engineering, Massachusetts Institute of Technology, Cambridge, 02139, USA

[2] Department of Aerospace and Mechanical Engineering, University of Notre Dame, Notre Dame, Indiana, 46556, USA

[3] Department of Materials Science and Engineering Department, Massachusetts Institute of Technology, Cambridge, 02139, USA

[4] Honda Research Institute, USA, Inc., San Jose, California 95134, USA

[#] Equal contribution

* Corresponding authors: Ju Li <liju@mit.edu>; Yanliang Zhang <yzhang45@nd.edu>

Materials

The substrate material used in this study for supporting nanoparticles is "Toray paper 060," bought from the Fuel Cell Store. The substrate size was 15 mm x 10 mm. The precursor used are Iron (II) acetate tetrahydrate, Nickel (II) acetate tetrahydrate, Cobalt (II) acetate tetrahydrate, Iron (III) Chloride hexahydrate, Nickel (II) Chloride hexahydrate, Cobalt (II) Chloride hexahydrate, Chromium (II) Chloride hexahydrate, Manganese (II) Chloride hexahydrate. All the precursors were bought from Sigma Aldrich.

Equipment

Pulsed light processing of nanoparticles uses Xenon 2100S flash lamp machine. This machine can generate high-power-density pulses (see schematic Fig. 1) of light covering the whole visible light spectrum. The xenon flash lamp works at very high voltages with a minimum of 1.9 kV and a maximum of 3.1 kV. The pulses of light can range from 100 microseconds to 3 milliseconds. The delay in Fig.1a is a function of the pulse voltage, pulse duration, and pulse number. The pulses occur in a closed box having control over the environment of reduction occurs. The sealed box contains a platform to hold the substrate distanced 2.5 cm from the lamp. The Xenon lamp produces a 3 inch x 3 inch of light footprint over the platform.

Scanning electron microscopy was performed in Notre Dame using a Helios G4 Ux Dual Beam Microscope. XPS was performed on Thermo K-Alpha+. STEM imaging was performed on Titan Themis Z G3 Cs-Corrected S/TEM.

The size measurement is performed using ImageJ software where brighter nanoparticles could be automatically separated from the darker background. The statistical analysis of the nanoparticle size is conducted with Origin software.

Method

In a typical process, separate 10 mM solutions of single metal salt in DI water are produced in glass vials with 15 minutes of stirring and 15 minutes of sonication. The solutions are then mixed in equal ratios to formulate a mixture of ternary, quaternary, and quinary precursor solution and sonicated again for 15 more minutes for uniform mixing. The prepared mixture is drop cast (200 µL) on a washed and oxygen plasma-treated substrate. A hot plate-assisted in-situ heating system is used to vaporize the water solvent and form a deposition of the precursor material on the substrate surface. Nafion is added on top of the precursor as binder to avoid detaching of the precursor materials from the substrate. The substrate is then transferred to the Xenon flash lamp machine for nanoparticle processing. Several light pulses generated by 3kV voltage with a 3ms duration were used for the high entropy alloy nanoparticle processing. The delay between two adjacent pulses was kept at 858ms. The whole procedure occurred in a closed conduit in less than 6 seconds of total time with a flowing nitrogen (N_2) atmosphere. The N_2 is used to avoid extensive oxidation of the nanoparticles at high temperature.

Flash Synthesis Parameter Selection

Flash synthesis parameters for all the samples were selected based on a separate study using a mixed chloride precursor of iron, nickel, and cobalt at equal ratio. The drop cast sample is irradiated with different pulse conditions from the Xenon flash lamp. The precursor's incomplete decomposition could be observed in the low-energy pulses (Figure S1 a-b). Uniform dispersion with sizes smaller than 100 nm could be found using voltage 3 kV, duration 3 ms, and 7 pulses with a delay between pulses of 858ms (Figure S1c). The theoretical maximum energy

deliverable at this state by the total seven pulses is estimated as 0.32 kJ/cm^2 Increasing pulse number beyond this parameter causes the nanoparticles to be welded together and become more prominent in size.

Electrochemical testing

All experiments were conducted in 1M KOH electrolyte. N_2 bubbling was performed for 45-60 min prior to the experiment to deaerate the solution from any oxygenated species. Hg/HgO was used as the reference electrode, enclosed in a plastic housing, to provide adequate robustness against strong alkaline environments. Catalyst activation was done by performing Cyclic Voltammetry (CV) at 20 and 50 mV/s scan rate. Linear sweep voltammetry (LSV) was conducted at 5 mV/s scan rate. Stability analysis was performed by applying constant current (i.e., Chronopotentiometry (CP)). CP was performed at different current densities (10, 50 and 200 mA/cm2), successively.

Table S1: Benchmarking of HEOH catalysts in comparison to other reported in literature

Catalyst	Over potential at 10 mA/cm2 (mV)	Stability (hr)	Ref
FeNiCo	400	10 hr @ 10mA/cm2	[1]
CoFeGaNiZn	370		[2]
FeNiCoCe	357	2hr @ 10 mA/cm2	[3]
AlNiCoFeMo	260	50 hr @10 mA/cm2	[4]
FeNiCoCr	221	20 hr @ 10 mA/cm2	[5]
CoCrFeNiMo	260	24 hr @ 100 mA/cm2	[6]
LaNiCoCrFeMn	325	50 hr @ 10 mA/cm2	[7]
**FeNiCoCr**	**250**	**50 hr @ 10 mA/cm2**<br>**+ 50 hr @ 50 mA/cm2**<br>**+ 20 hr @ 250 mA/cm2**	**This work**

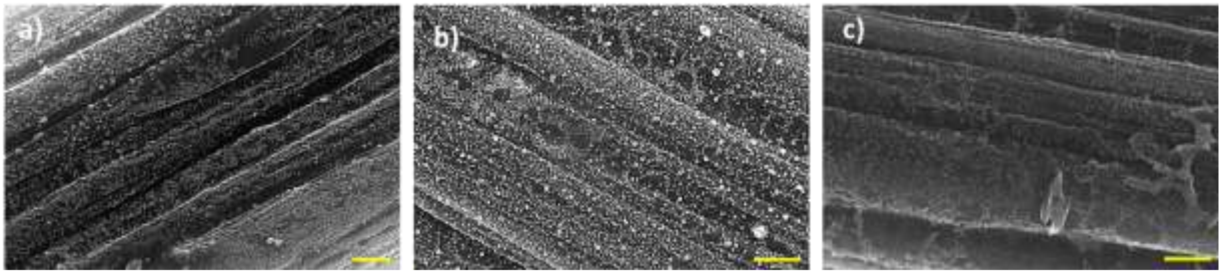

Figure S1 *Nanoparticle coverage after pulsed light synthesis a) HEO(FeNiCo)$_{Cl}$, b) HEO(FeNiCoCr)$_{Cl}$, c) HEO(FeNiCoCrMn)$_{Cl}$ (scale bar 500 nm).*

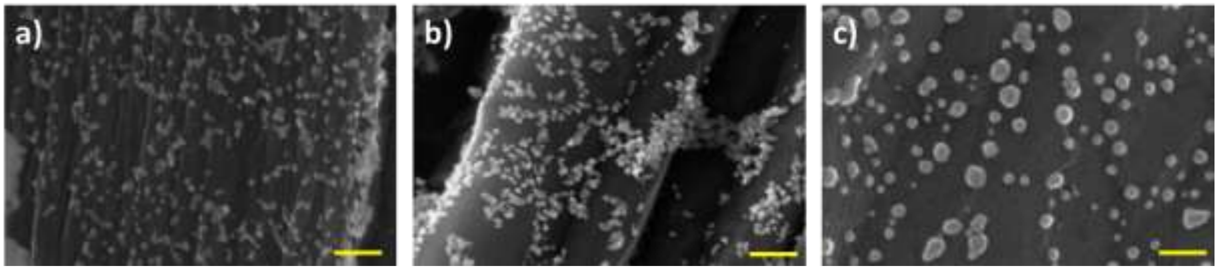

Figure S2 *SEM images of a) Ternary HEO(FeNiCo)$_{Cl}$, b) Quaternary HEO(FeNiCoCr)$_{Cl}$, and c) Quinary HEO(FeNiCoCrMn)$_{Cl}$ alloy nanoparticles (scale bar 100 nm).*

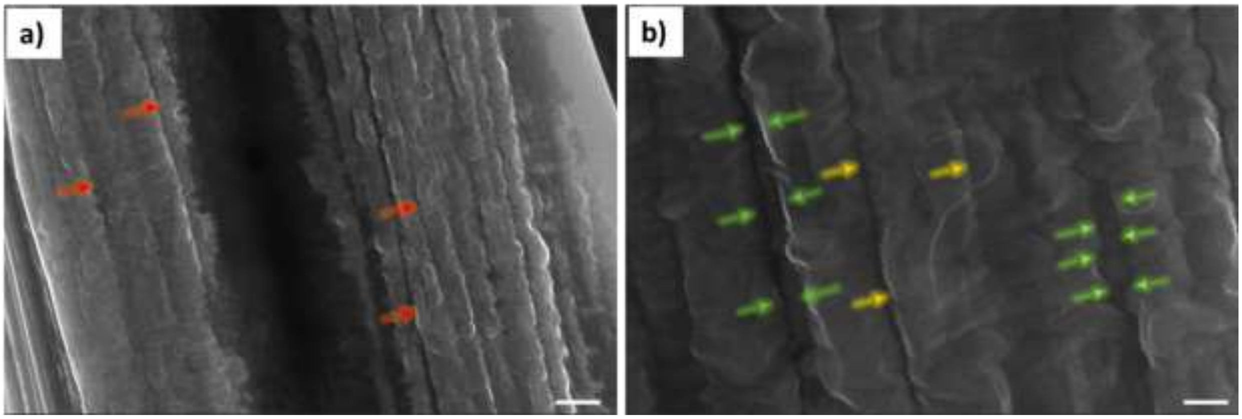

Figure S3 *a-b) Plasma cleaned substrate. Yellow arrows denote small wrinkle-like and green arrows denoting larger crater-like defects*

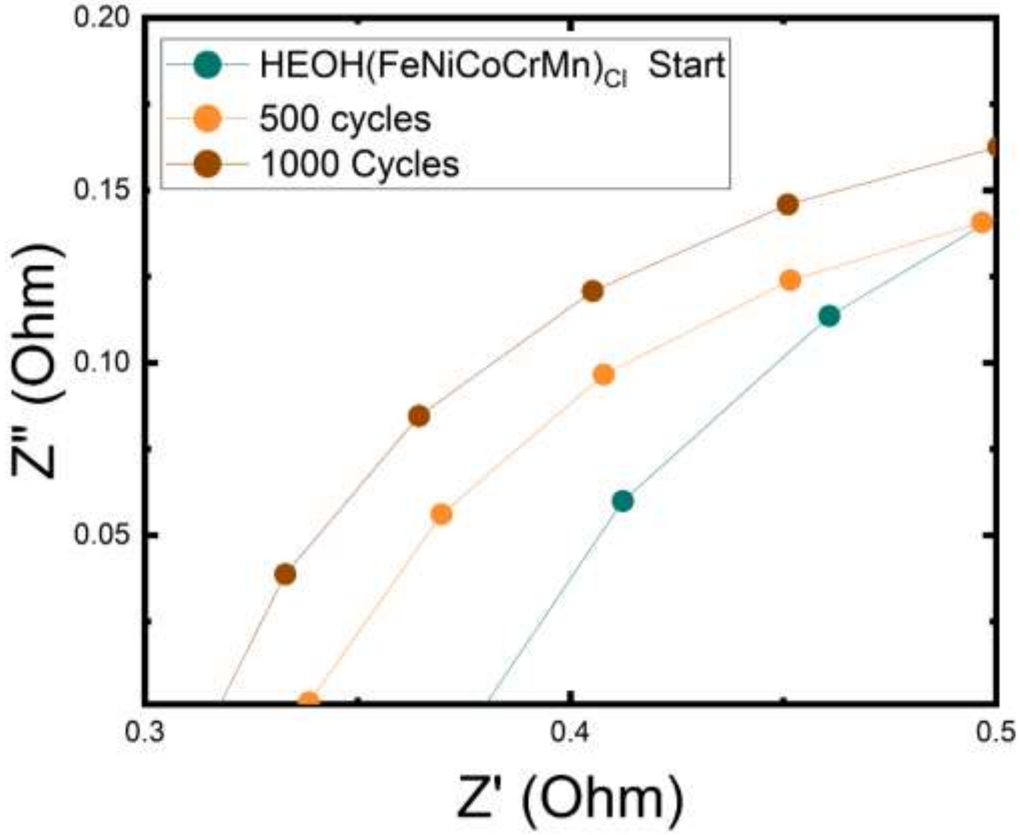

Figure S4: *EIS data of HEOH(FeNiCoCrMn)$_{Cl}$ before cycling (green), and after 500 and 1000 cycles (orange and brown, respectively). Intercepts of the x-axis represent the ohmic resistance portion, where ohmic resistance is reduced with cycling.*

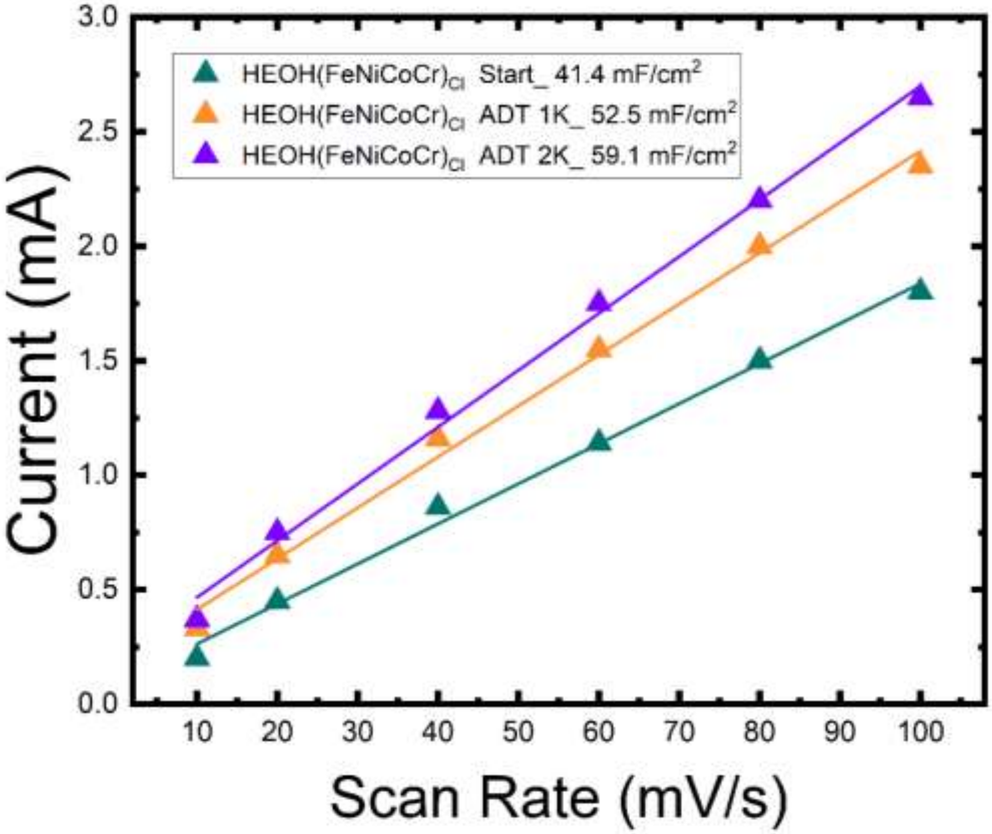

Figure S5: *ECSA trends at different cycles showing monotonic enhancement of ECSA with cycling for HEOH(FeNiCoCr)$_{Cl}$*

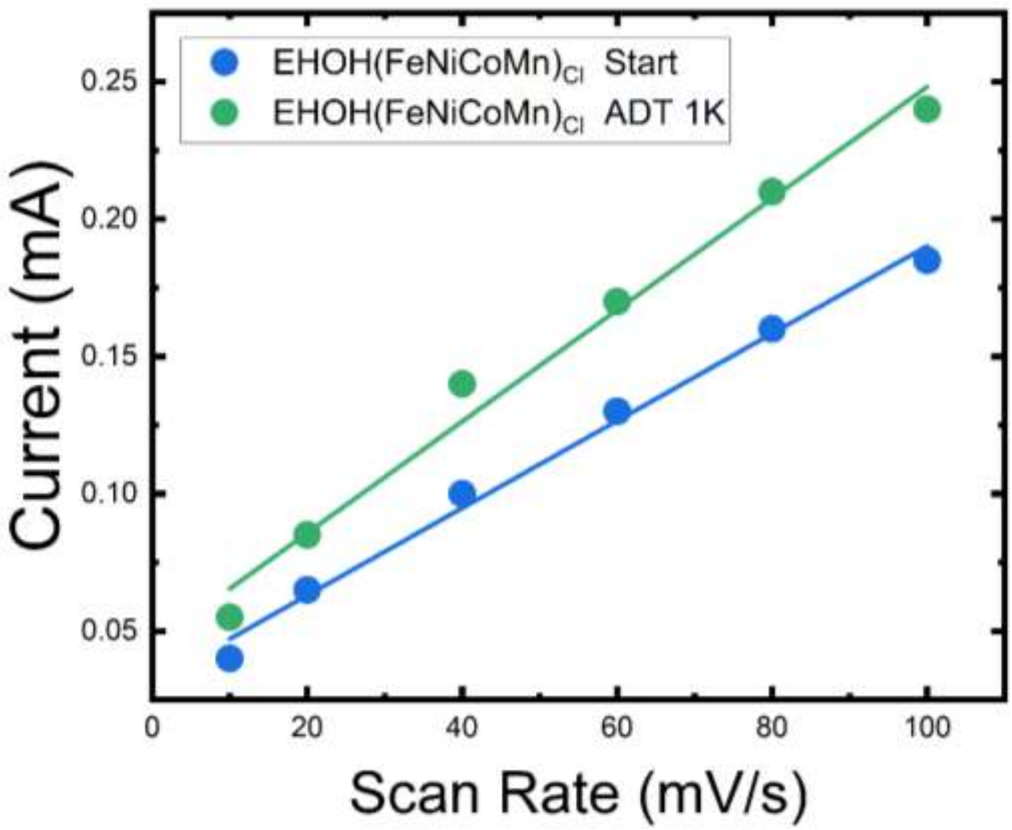

Figure S6: *ECSA trends at different cycles showing monotonic enhancement of ECSA with cycling for HEOH(FeNiCoMn)$_{Cl}$*

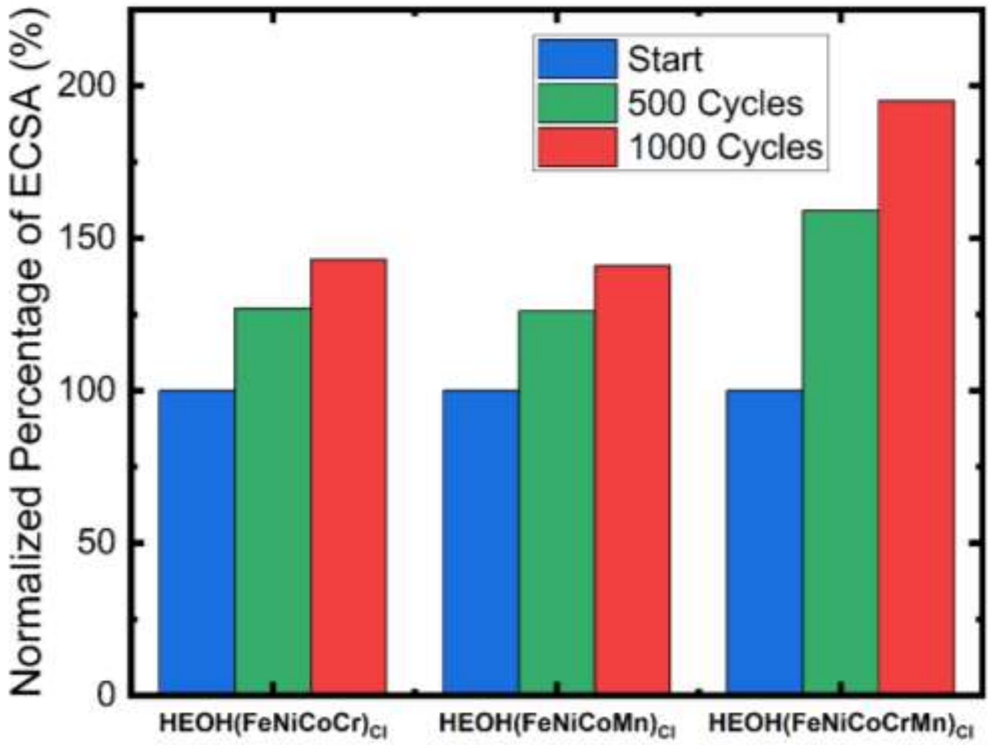

Figure S7: *Bar chart comparing ECSA evolution of different samples during cycling*

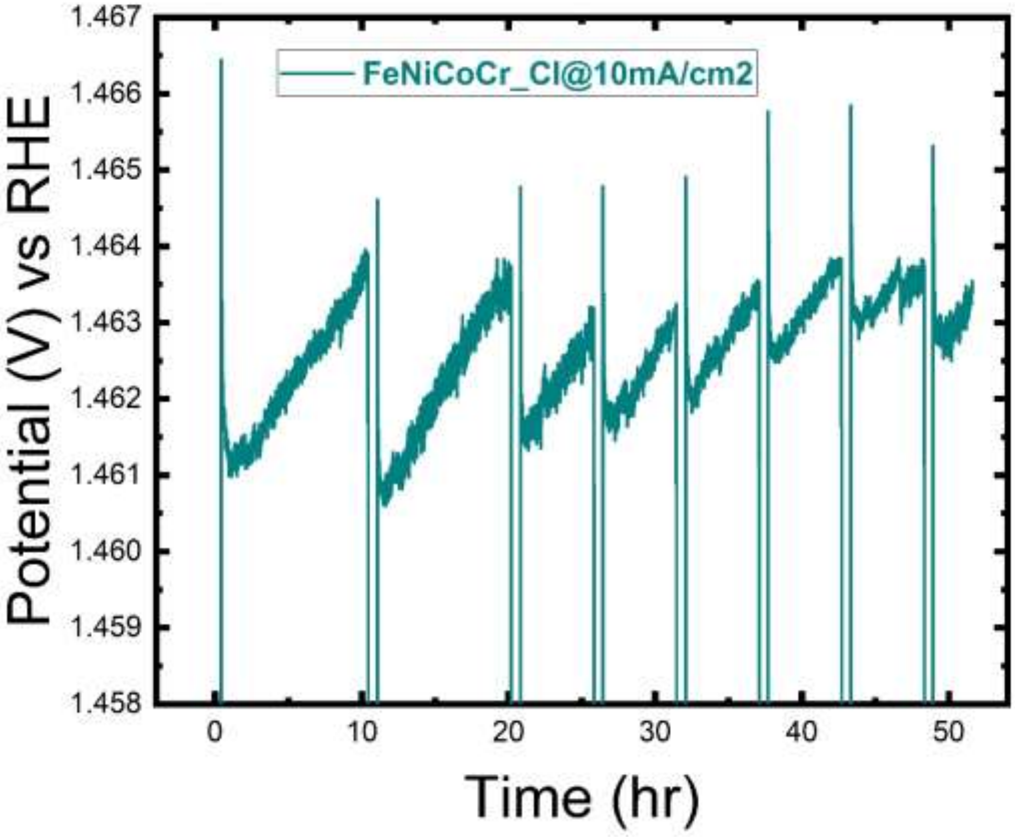

Figure S8: *Chronopotentiometry (CP[10]) ADT of HEOH(FeNiCoCr)$_{Cl}$ at 10 mA/cm^2 for 50 hrs*

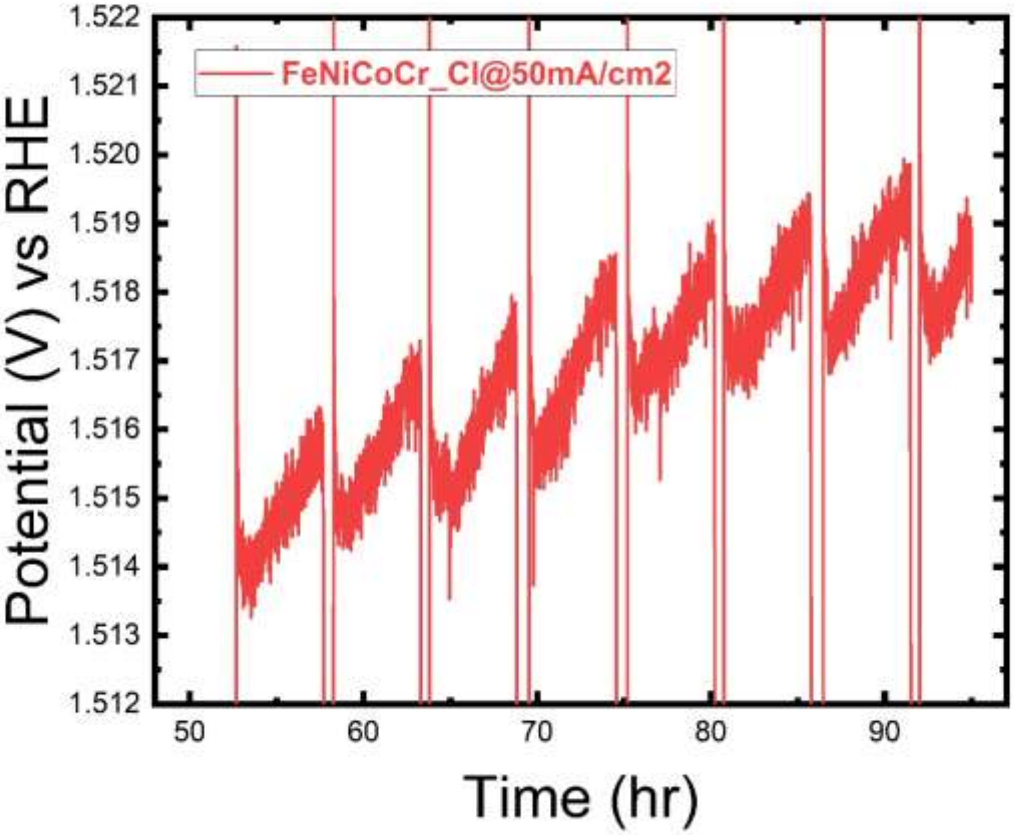

Figure S9: *Chronopotentiometry (CP[50]) ADT of HEOH(FeNiCoCr)$_{Cl}$ at 50 mA/cm^2 for 50 hrs*

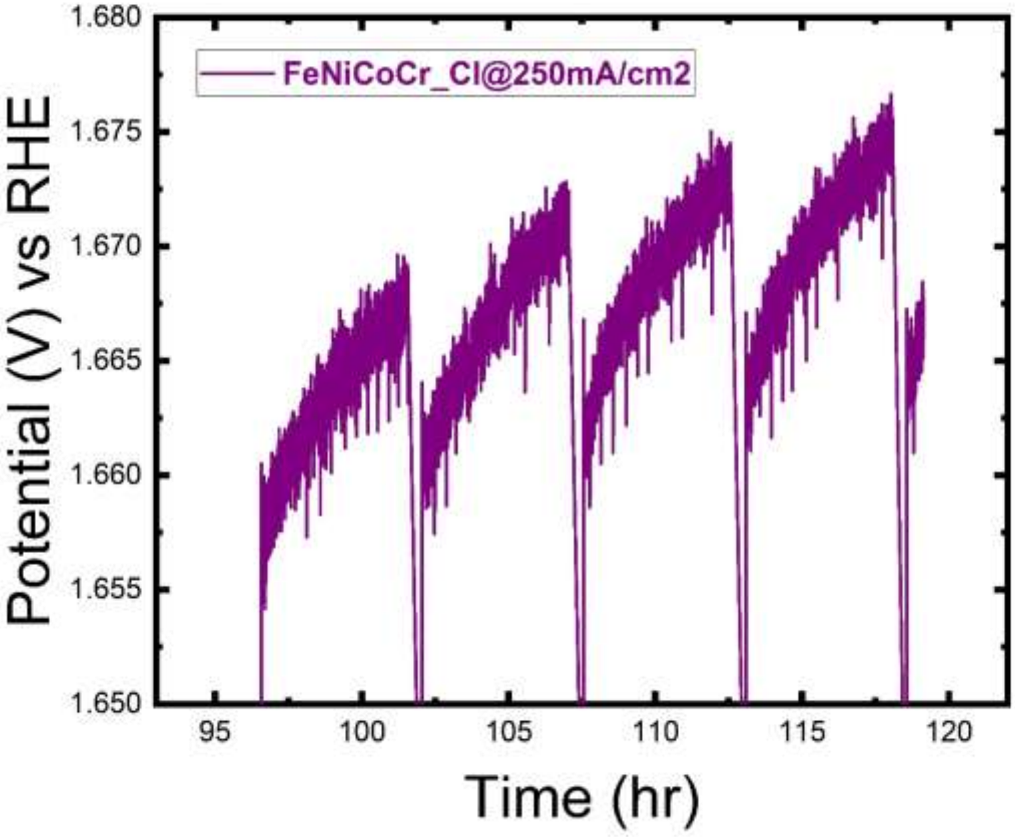

Figure S10: *Chronopotentiometry (CP^{250}) ADT of HEOH(FeNiCoCr)$_{Cl}$ at 250 mA/cm^2 for 20 hrs*

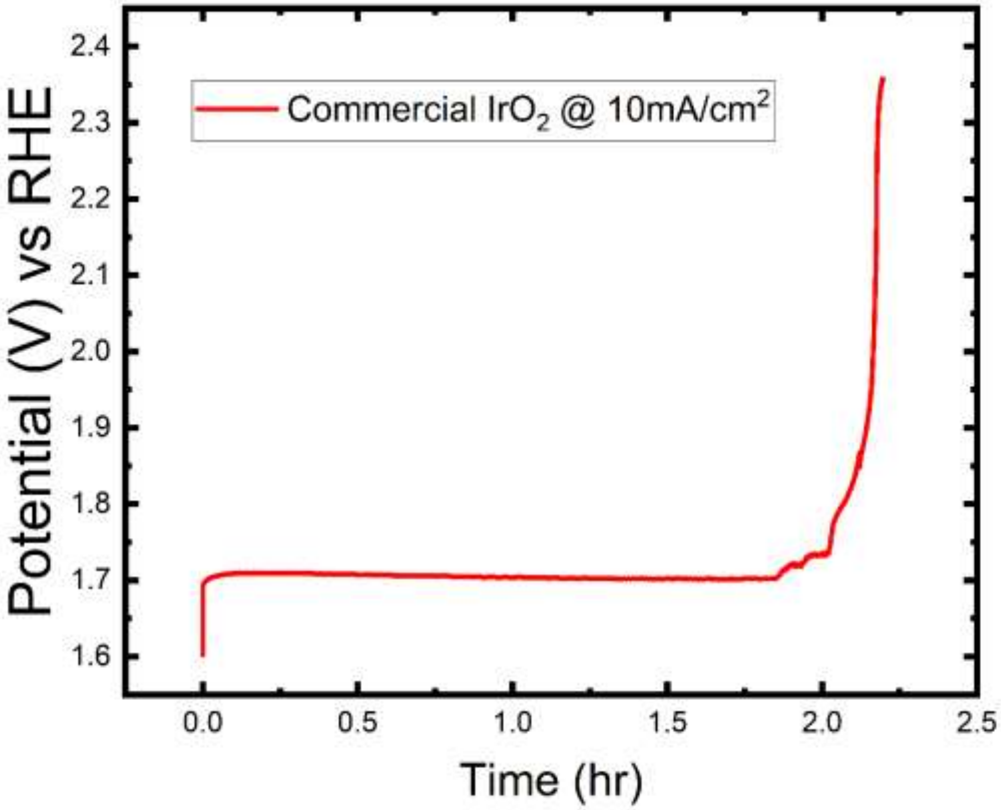

Figure S11: *Chronopotentiometry (CP*[10]*) ADT of commercial IrO_2*

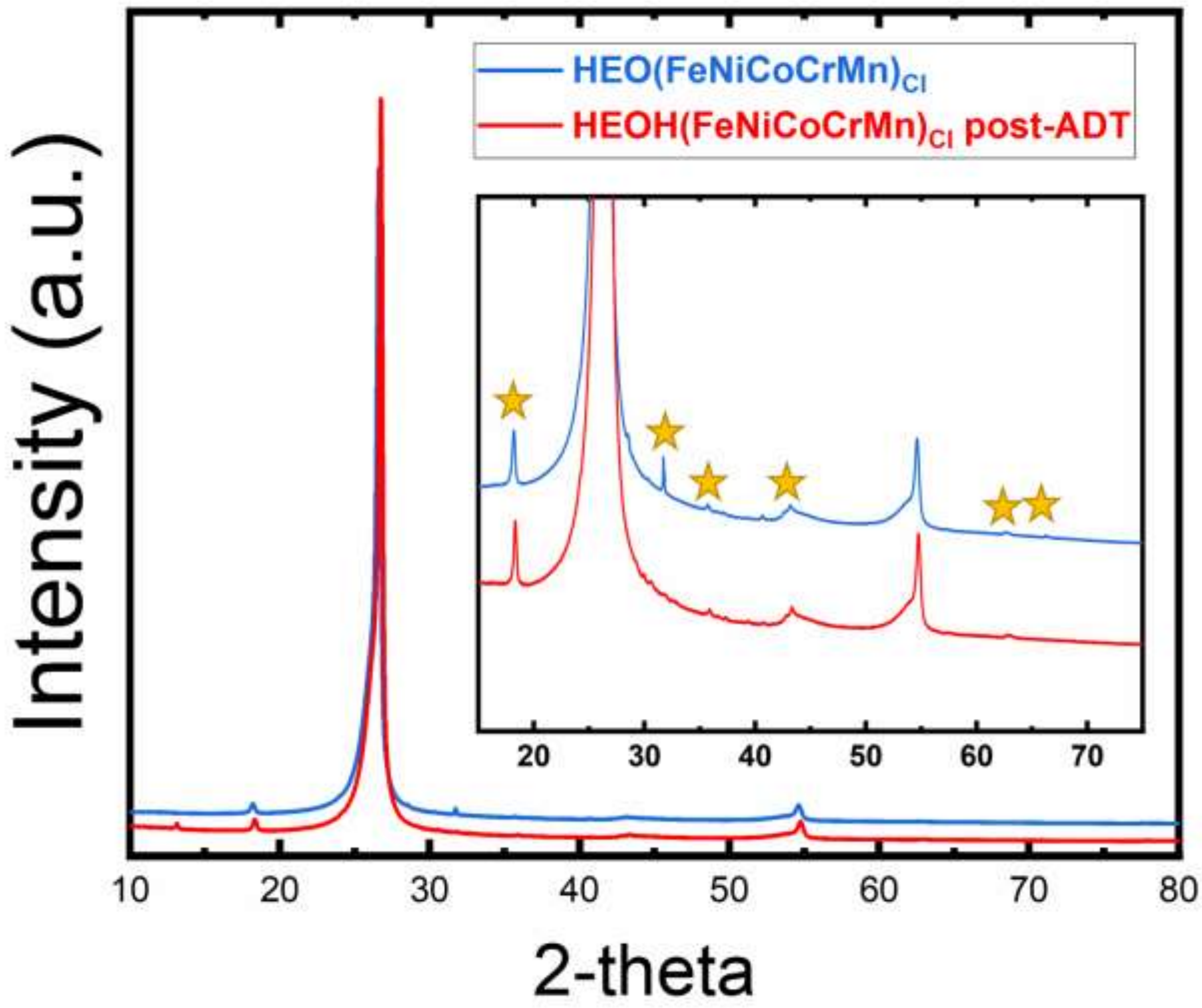

Figure S12: XRD Spectra before and after accelerated durability testing (ADT)

References

[1] S. Saha, A. K. Ganguli, **n.d.**, DOI 10.1002/slct.201601243.

[2] L. Sharma, N. Kumar Katiyar, A. Parui, R. Das, R. Kumar, C. S. Tiwary, A. K. Singh, A. Halder, K. Biswas, *Nano Res* **2022**, 4799.

[3] J. A. Haber, Y. Cai, S. Jung, C. Xiang, S. Mitrovic, J. Jin, A. T. Bell, J. M. Gregoire, *Cite this: Energy Environ. Sci* **2014**, *7*, 682.

[4] H. J. Qiu, G. Fang, J. Gao, Y. Wen, J. Lv, H. Li, G. Xie, X. Liu, S. Sun, *ACS Mater Lett* **2019**, *1*, 526.

[5] N. Zhang, X. Feng, D. Rao, X. Deng, L. Cai, B. Qiu, R. Long, Y. Xiong, Y. Lu, Y. Chai, *Nature Communications 2020 11:1* **2020**, *11*, 1.

[6] Z. Chen, K. Huang, T. Zhang, J. Xia, J. Wu, Z. Zhang, B. Zhang, *Processes* **2023**, *11*, 245.

[7] T. X. Nguyen, Y. C. Liao, C. C. Lin, Y. H. Su, J. M. Ting, *Adv Funct Mater* **2021**, *31*, DOI 10.1002/ADFM.202101632.
